# A Low Frequency FBG Accelerometer with Symmetrical Bended Spring Plates

**DOI:** 10.3390/s17010206

**Published:** 2017-01-22

**Authors:** Fufei Liu, Yutang Dai, Joseph Muna Karanja, Minghong Yang

**Affiliations:** 1 National Engineering Laboratory for Fiber Optic Sensing Technology, Wuhan University of Technology, Luoshi Road 122, 430070 Wuhan, China; liufufei_2000@126.com; 2 Physics Department, Pwani University, P.O. Box 195-80108 Kilifi, Kenya; kamuna2007@yahoo.com

**Keywords:** fiber optics sensor, accelerometer, low frequency, vibration monitoring

## Abstract

To meet the requirements for low-frequency vibration monitoring, a new type of FBG (fiber Bragg grating) accelerometer with a bended spring plate is proposed. Two symmetrical bended spring plates are used as elastic elements, which drive the FBG to produce axial strains equal in magnitude but opposite in direction when exciting vibrations exist, leading to doubling the wavelength shift of the FBG. The mechanics model and a numerical method are presented in this paper, with which the influence of the structural parameters on the sensitivity and the eigenfrequency are discussed. The test results show that the sensitivity of the accelerometer is more than 1000 pm/g when the frequency is within the 0.7–20 Hz range.

## 1. Introduction

Vibration measurement is an important and key issue in modern engineering applications. Traditional accelerometers are usually the piezoelectric type and piezo-resistive type, etc. [1,2]. However, these sensors, when applied on a large scale or applied in flammable explosive environments, can be affected by electromagnetic interference, and may even develop a risk of inflammation. To address these problems, the use of optical technology in the design of accelerometers is needed, in contrast to the traditional electronic ones [3].

Over the last two decades, FBG (fiber Bragg grating)-based accelerometers have attracted much more attention from both researchers and engineers. Many advantages of accelerometers based on FBG, such as immunity to electromagnetic radiation, resistance to chemical corrosion, small size, high accuracy, capability of remote operation [4,5], low noise and multiplexing capabilities, have been discovered in previous studies. These characteristics promote the application of FBG accelerometers in remote online monitoring and measurement in harsh environments [6,7,8], for instance in earthquakes, debris flow, oil and gas, wind energy, coal mines, and even nuclear explosions [9,10]. In recent years, great progress has been made in the design of FBG-based accelerometers, such as the embedded type and cantilevers [11,12,13,14,15], accelerometers based on a diaphragm [16,17]. Among the various types of FBG accelerometers, Zhang et al. reported an accelerometer that provided an extremely high sensitivity of 1296 pm/g and a frequency range from 0 to 25 Hz [14]. However, due to the use of two FBGs, demodulation became more difficult. In [15], Basumallick proposed an accelerometer with a sensitivity of 1062 pm/g with the range of frequency being only 10 Hz. However, when the frequency was raised to 25 Hz, its sensitivity declined to only 20.7 pm/g with the 1000 μm patch or 7.8 pm/g without any patch. Liu presented an FBG accelerometer based on a single diaphragm and on double diaphragms [16,17]. The former had a broad frequency ranging from 10 to 200 Hz, but a low sensitivity of 36.6 pm/g [16]. The latter provided a broader frequency range from 50 to 800 Hz with corresponding sensitivity ranging from 23.8 to 45.9 pm/g [17].

Generally, there is a limit of available frequency for most FBG-based accelerometers. Stefani et al. [18] proposed a polymer optical fiber Bragg grating–based accelerometer for operation at both 1550 and 850 nm. Based on our research [19], however, for those accelerometers with a higher resonance frequency, their low frequency response would be not good, especially when the work frequency is lower than 15 Hz. Therefore, it is necessary to develop an accelerometer that has high sensitivity and can satisfy the requirements for lower-frequency applications.

This paper, aiming to monitor low-frequency vibrations, demonstrates a new type of FBG accelerometer with bended elastic plates. The symmetrical arrangement of the bended spring plates can make the fiber Bragg grating produce a tensile strength or compression equal in magnitude in the opposite direction. Therefore, the sensitivity of the sensor can be nearly doubled. The mechanics model of the sensor is presented. By use of the numerical method, we explore the influence of the structural parameters on the sensitivity and the eigenfrequency. A sensor design example and its test results are experimentally demonstrated.

## 2. Principle and Structure of the Sensor

**Figure 1:**
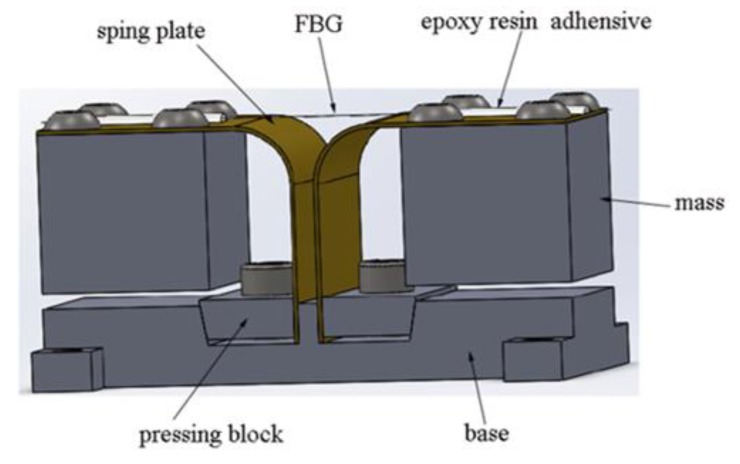
The schematic illustration of the accelerometer.

The proposed low-frequency accelerometer is based on FBG and a novel mass-spring plate system, in which two symmetrical bended spring plates are used as elastic elements. The schematic illustration of the accelerometer is shown in Figure 1.

Obviously, the composition of the sensor bulk is considerably simple, and it consists of a fiber grating, bended spring plates, inertia masses, pressing blocks, the base and some connecting parts. The bended spring plate is made of bent and molded beryllium bronze. The plate is composed of three sections, including the vertical section, the bended section and the horizontal section. The inertia mass is connected with the horizontal section by screws. The vertical section is fixed in the base with the pressing block. As elastic elements, the spring plates play an important role in transforming the vertical acceleration of the vibration exciter to the axial strain of the FBG. Two ends of the fiber grating are pasted on the surface of the horizontal section of the bended spring plate with epoxy resin. The Bragg grating is suspended and aligned on the sensor. Such an arrangement can effectively avoid the chirped phenomenon by the inhomogeneous strain in the working process. Four through-holes are machined at the four corners of the base, which are used to fix the sensor on the detected object. Due to the design and selection of the construction materials, the accelerometer has achieved higher reliability at lower cost.

Under the condition of ambient vibration, the accelerometer will vibrate with the excited frequency synchronously. Therefore, the two symmetrical inertia masses will produce micro-vibrations and a swing angle around the respective spring plates. Meanwhile, the ends of the FBG will produce counter-deformations in its axial direction which yields resultant double deformations due to the amplitude superposition effect, hence doubling its sensitivity approximately.

## 3. Mathematical Model and Calculations

**Figure 2:**
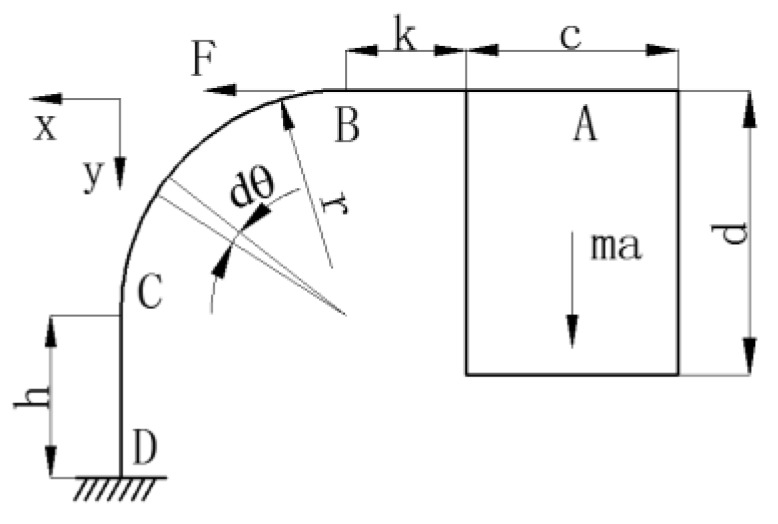
Simplified structure diagram of the sensor and the meaning of each symbol. x, y: the horizontal and vertical directions; *F*: force at point B; *h*: the height of CD segment; *r*: the radius of the bended segment; *k*: the length of horizontal segment; *m*: mass; *c*, *d*: the length and height of the mass; *a*: the exciting acceleration; *dθ*: the infinitesimal angle.

To obtain the ideal performance of the accelerometer, i.e., to balance the higher sensitivity with the proper eigenfrequency, it is necessary to optimize the dimensions of the modular construction. In order to calculate the sensitivity of the acceleration sensor, we need to get the amount of tension of the FBG under the action of the inertial force. Considering the symmetrical structure of the sensor, to calculate it simply and easily, we only take the single side of the sensor. Firstly, simplifying the structure of the sensor, we can get the following structure diagram as shown in Figure 2. The dθ is the infinitesimal angle. The x, y represent the horizontal and vertical directions, respectively.

Utilizing the superposition principle and unit load method [20], through the superposition of all slight displacements at point B, the following balance equations were obtained:

(1) $$\{\begin{array}{c} \begin{array}{c} \Delta_{BH}=\frac{1}{EI}\{\int_{0}^{\frac{\pi }{2}}\left[ma\left(l+r\sin\theta \right)-Fr\left(1-\cos\theta \right)\right]\cdot r\left(1-\cos\theta \right)rd\theta \\ +\int_{0}^{h}\left[ma\left(l+r\right)-F\left(r+y\right)\right]\cdot \left(r+y\right)dy\} \end{array}\\ F=\frac{E_{f}A_{f}}{l_{f}}\Delta_{BH} \end{array}$$

where ∆*_BH_* is the horizontal displacement of point B; *E* is the elastic modulus of beryllium bronze, which is the material of the bended spring plate; *I* is the cross-section inertia moment of the bended spring plate; *I* = *bt*^3^/12, *b* is the width of the plate; *t* is thickness of the plate; *m* = *ρbcd*; *ρ* is the density of the mass, and the mass is made of the ordinary carbon steel; *c* is the length of the mass; *d* is the height of the mass; *a* is the exciting acceleration; *l* = *k* + *c*/2; *k* is the length of the horizontal segment; *r* is the radius of the bended segment; *h* is the height of the vertical segment; *F* is the horizontal force of the fiber at point B; *E_f_* is the elastic modulus of the fiber materials; *A_f_* is the cross-section area of the fiber; *l_f_* is the distance from point B to the center of the FBG. Based on the simultaneous Equation (1), we can get:

(2) $$\Delta_{BH}=\frac{6mal_{f}\left[r^{2}\left(r-2l+\pi l\right)+h\left(l+r\right)\left(h+2r\right)\right]}{12EIl_{f}+E_{f}A_{f}\left[3r^{3}\left(3\pi -8\right)+12r^{2}h+12rh^{2}+4h^{3}\right]}$$

Taking the symmetrical nature of the structure into account, the stretching amount of the FBG should be two times the horizontal displacement of point B:

(3) $$\delta =2\Delta_{BH}$$

So, the center wavelength shift of the FBG in the sensor can be expressed as the following equation:

(4) $$\Delta \lambda =\left(1-P_{e}\right)\lambda \frac{\delta }{2l_{f}}=\left(1-P_{e}\right)\lambda \frac{\Delta_{BH}}{l_{f}}$$

where *P_e_* is elasto-optical coefficient. In general, the elasto-optical coefficient of the germanium-doped silica fiber is 0.22. So, we get the sensitivity of the sensor:

(5) $$S=\frac{\Delta \lambda }{a}=\left(1-P_{e}\right)\lambda \frac{6\rho bcd\left[r^{2}\left(r-2l+\pi l\right)+h\left(l+r\right)\left(h+2r\right)\right]}{12EIl_{f}+E_{f}A_{f}\left[3r^{3}\left(3\pi -8\right)+12r^{2}h+12rh^{2}+4h^{3}\right]}$$

On the other hand, the eigenfrequency of the accelerometer is also an important factor that can affect the available measurement frequency range. Only considering the vibration in the vertical direction, it can be seen that the sensor has two degrees of freedom in the vertical direction by observing the structure of the sensor. The two-degree-freedom system has two different resonant frequencies. We will solve the resonant frequency in the vertical direction of the sensor using the compliance method.

At any moment in the process of free vibration, for each degree of freedom, the displacement of the barycenter of the mass should be equal to the static displacement of the vibration system under the action of the inertia force at that time. Accordingly, we can establish the following vibration equation:

(6) $$\{\begin{array}{c} \delta_{11}m\omega^{2}\Delta x+\delta_{12}m\omega^{2}\Delta y=\Delta x\\ \delta_{21}m\omega^{2}\Delta x+\delta_{22}m\omega^{2}\Delta y=\Delta y \end{array}$$

where *δ*_11_ is the horizontal displacement of the barycenter of the mass under the action of the unit horizontal force, *δ*_12_ is the horizontal displacement of the barycenter under the action of the unit vertical force, *δ*_21_ is the vertical displacement of the barycenter under the action of the unit horizontal force, *δ*_22_ is the vertical displacement of the barycenter under the action of the unit vertical force, *ω* is the angular velocity of the barycenter, ∆*x* is the horizontal displacement of the mass center under the inertia force, ∆*y* is the vertical displacement of the barycenter under the inertia force.

In Equation (6) above, the parameters *δ*_11_, *δ*_21_, *δ*_12_ and *δ*_22_ can be obtained by the unit load method [18] and the superposition principle. Because the derived procedure of these parameters is very complex, omitting the intermediate procedure, we can get:

(7) $$\begin{array}{ll} \delta_{11} & =\frac{1}{EI}{\{\int_{0}^{k}\left(\frac{d}{2}\right)}^{2}dx+\int_{0}^{\frac{\pi }{2}}\left[-\frac{d}{2}+r\left(1-\cos\theta \right)-F_{1}r\left(1-\cos\theta \right)\right]\left[-\frac{d}{2}+r\left(1-\cos\theta \right)\right]rd\theta \\ & +\int_{0}^{\frac{d}{2}-r}\left[-\left(\frac{d}{2}-r-x\right)-F_{1}\left(r+x\right)\right]\left[-\left(\frac{d}{2}-r-x\right)\right]dx+\int_{0}^{r+h-\frac{d}{2}}\left[x-F_{1}\left(\frac{d}{2}+x\right)\right]dx\} \end{array}$$

(8) $$\begin{array}{ll} \delta_{21} & =\frac{1}{EI}\{\int_{0}^{k}\left(-\frac{d}{2}\right)\left(\frac{c}{2}+x\right)dx+\int_{0}^{\frac{\pi }{2}}\left[-\frac{d}{2}+r\left(1-\cos\theta \right)-F_{1}r\left(1-\cos\theta \right)\right]\cdot \left(l+r\sin\theta \right)rd\theta \\ & +\int_{0}^{\frac{d}{2}-r}\left[-\left(\frac{d}{2}-r-x\right)-F_{1}\left(r+x\right)\right]\cdot \left(l+r\right)dx+\int_{0}^{r+h-\frac{d}{2}}\left[x-F_{1}\left(\frac{d}{2}+x\right)\right]\left(l+r\right)dx\} \end{array}$$

(9) $$\begin{array}{ll} \delta_{12} & =\frac{1}{EI}\{\int_{0}^{k}\left(\frac{c}{2}+x\right)\left(-\frac{d}{2}\right)dx+\int_{0}^{\frac{\pi }{2}}\left[l+r\sin\theta -F_{2}r\left(1-\cos\theta \right)\right]\cdot \left[-\frac{d}{2}+r\left(1-\cos\theta \right)\right]rd\theta \\ & +\int_{0}^{\frac{d}{2}-r}\left[l+r-F_{2}\left(r+x\right)\right]\cdot \left(r-\frac{d}{2}+x\right)dx+\int_{0}^{r+h-\frac{d}{2}}\left[l+r-F_{2}\left(\frac{d}{2}+x\right)\right]\cdot xdx\} \end{array}$$

(10) $$\begin{array}{ll} \delta_{22} & =\frac{1}{EI}\{\int_{0}^{k}{\left(\frac{c}{2}+x\right)}^{2}+\int_{0}^{\frac{\pi }{2}}\left[l+r\sin\theta -F_{2}r\left(1-\cos\theta \right)\right]\cdot \left(l+r\sin\theta \right)rd\theta \\ & +\int_{0}^{h}\left[l+r-F_{2}\left(r+x\right)\right]\cdot \left(l+r\right)dx\} \end{array}$$

where *F*_1_ is the pulling force of the FBG under the action of the unit horizontal load, *F*_2_ is the pulling force of the FBG under the action of the unit vertical load. They can be calculated by:

(11) $$F_{1}=\frac{E_{f}A_{f}\left[\left(d+4h+4r\right){\left(2h-d+2r\right)}^{2}-\left(d+4r\right){\left(d-2r\right)}^{2}+12r^{2}\left(2d-8r-\pi d+3\pi r\right)\right]}{48EIl_{f}+2E_{f}A_{f}\left[8h^{3}+24h^{2}r+24hr^{2}+6r^{3}\left(3\pi -8\right)\right]}$$

(12) $$F_{2}=\frac{6E_{f}A_{f}\left[r^{2}\left(r-2l+\pi l\right)+h\left(l+r\right)\left(h+2r\right)\right]}{12EIl_{f}+E_{f}A_{f}\left[3r^{3}\left(3\pi -8\right)+4h^{3}+12h^{2}r+12hr^{2}\right]}$$

In order to get the solution of ∆*x* and ∆*y* which are not both equal to zero, the coefficient determinant of Equation (6) above should be equal to zero, as in:

(13) $$D=\left|\begin{array}{cc} m\omega^{2}\delta_{11}-1 & m\omega^{2}\delta_{12}\\ m\omega^{2}\delta_{21} & m\omega^{2}\delta_{22}-1 \end{array}\right|=0$$

Expanding the above equation, we can get the frequency equation expressed by:

(14) $$\left(\delta_{11}\delta_{22}-\delta_{12}\delta_{21}\right)m^{2}\omega^{4}-\left(\delta_{11}+\delta_{22}\right)m\omega^{2}+1=0$$

Solving the frequency equation, the two roots about ω^2^ are obtained:

(15) $${\omega_{1,2}}^{2}=\frac{\left(\delta_{11}+\delta_{22}\right)m\mp \sqrt{{\left(\delta_{11}-\delta_{22}\right)}^{2}m^{2}+4\delta_{12}\delta_{21}m^{2}}}{2m^{2}\left(\delta_{11}\delta_{22}-\delta_{12}\delta_{21}\right)}$$

The two eigenfrequencies of the sensor in the vertical direction can be calculated by:

(16) $$f_{1}=\frac{\omega_{1}}{2\pi },\;f_{2}=\frac{\omega_{2}}{2\pi }$$

## 4. Optimization Design of the Parameters

In order to obtain the maximum sensitivity of the sensor, while the eigenfrequency is in an ideal range, it is necessary to perform design optimization for the structure parameters. Of course, the structure size must be given a reasonable restricted range. Therefore, the objective function of the optimized model solves the maximum sensitivity of the sensor as follows:

(17) $$max\;S=\left(1-P_{e}\right)\lambda \frac{6\rho bcd\left[r^{2}\left(r-2l+\pi l\right)+h\left(l+r\right)\left(h+2r\right)\right]}{12EIl_{f}+E_{f}A_{f}\left[3r^{3}\left(3\pi -8\right)+12r^{2}h+12rh^{2}+4h^{3}\right]}$$

Then, some constraint conditions must be considered. From Equations (15) and (16), there are two eigenfrequencies for the sensor. We define the smaller eigenfrequency as *f_m_*. For ensuring the sensor works in a lower frequency range, we can restrict the value of *f_m_* to the range of 50 to 60 Hz. Additionally, structure size of the sensor should be controlled in a relatively reasonable range. For example, the total length of the sensor is restricted to less than 50 mm, the total height is restricted to within 30 mm, the space between the bottom surface of the mass and the top surface of the base cannot be less than 2 mm, and so on. Finally, the constraint conditions can be expressed as:

(18) $$s.t.\{\begin{array}{c} 50\leq f_{m}\leq 60\\ r+c+k\leq 25\\ h+r\leq 25\\ h+r-d\geq 2\\ 15\leq b\leq 20\\ 10\leq c\leq 30\\ 2\leq k\leq 10\\ 6\leq r\leq 20\\ 0\leq h\leq 20\\ 10\leq d\leq 23 \end{array}$$

According to the objective function and the constraint conditions, the optimized mathematical model can be established. In addition, the optimization program was compiled in the “LINGO (Linear Interactive and General Optimizer)” approach. Then, using the global solver, the optimal solution of the sensitivity under constraint conditions was obtained as 851 pm/g, while fm was 50 Hz. Considering the convenience of manufacturing the sensor, the optimized final structural parameters were rounded off as follows: *c* = 17 mm, *b* = 20 mm, *d* = 16 mm; *k* = 3 mm, *r* = 6 mm, *h* = 12 mm; the space between two bended spring plates is 2 mm.

Substituting the rounded-off parameters into the sensitivity and the eigenfrequency formula, the theoretical sensitivity becomes 877.2 pm/g, while the first- and second-order theoretical values of the eigenfrequency are 51.1378 Hz and 235.0104 Hz, respectively.

**Figure 3:**
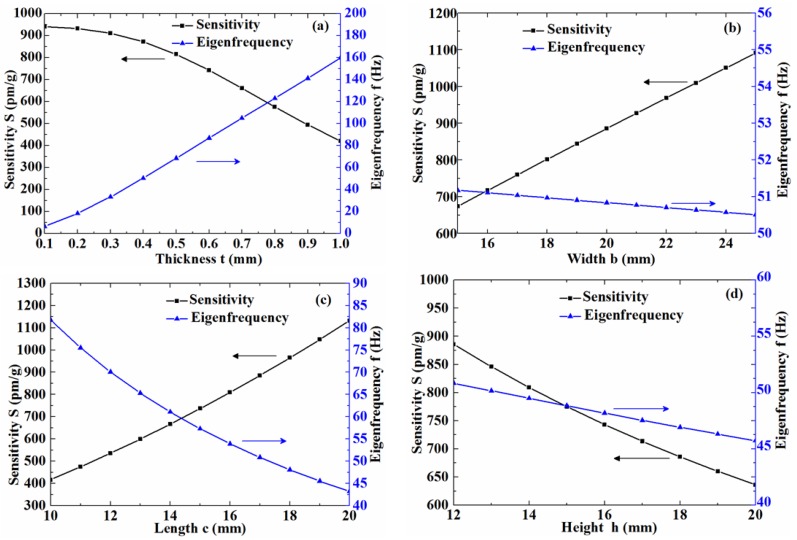
The influence of major parameters on the sensitivity and eigenfrequency. The influence of thickness *t* (**a**); the influence of width *b* (**b**); the influence of length *c* (**c**); the influence of height *h* (**d**).

Subsequently, we will discuss the influence of the major parameters (*t*, *b*, *c*, *h*) on the sensitivity and the eigenfrequency. It is known from Equation (5) that the plate thickness *t* directly affects the inertia moment *I*; thus, it has great influence on the sensitivity. Based on Equations (5), (15) and (16), the sensitivity *S* and the eigenfrequency *f* can be calculated using MATLAB software. By varying only one parameter of *t*, *b*, *c* and *h*, and fixing the other parameters (*t*, *b*, *c*, *h*, *d*, *r*, *k*), we can get the influence curves of the major parameters, as shown in Figure 3.

From Figure 3a, when *t* was 0.1~0.4 mm, S was about 900 pm/g, with very small change. When *t* increased gradually from 0.4 mm to 1 mm, S reduced greatly, from 900 pm/g to 400 pm/g. When *t* increased gradually from 0.1 mm to 1 mm, *f* increased significantly, from 10 Hz to 160 Hz. It can be seen from the above analysis that the thickness *t* has a great influence on the value of *S* and *f*, and reducing the thickness can improve the sensitivity and lower the eigenfrequency. However, taking into account the structural strength of the sensor, which ensures better transverse anti-interference ability, the thickness should not be too thin. Usually, we set *t* = 0.4 mm for our sensor.

Figure 3b shows the influences of the width *b* on the sensitivity and the eigenfrequency. When *b* increased gradually from 15 mm to 25 mm, the sensitivity rose substantially from 700 pm/g to 1100 pm/g; the eigenfrequency changed very little, remaining around 51 Hz. Based on the above analysis, it is known that increasing the width can significantly improve the sensitivity, but it has little influence on the eigenfrequency.

The influence curves of the length *c* on the sensitivity and the eigenfrequency are shown in Figure 3c. When *c* increased gradually from 10 mm to 20 mm, the sensitivity rose sharply from 400 pm/g to 1100 pm/g, and the eigenfrequency decreased greatly, from 80 Hz down to 45 Hz. From the above analysis, it is known that increasing the length *c* can improve greatly the sensitivity and lower the eigenfrequency.

The influence of the curves of the height *h* on the sensitivity and the eigenfrequency are shown in Figure 3d. When *h* increased gradually from 12 mm to 20 mm, the sensitivity decreased rapidly from 880 pm/g to around 640 pm/g, and the eigenfrequency decreased from 50.5 Hz down to 46 Hz. Based on the above analysis, it is known that reducing the height *h* can significantly improve the sensitivity and slightly increase the eigenfrequency.

## 5. Experimental Results and Discussion

**Figure 4:**
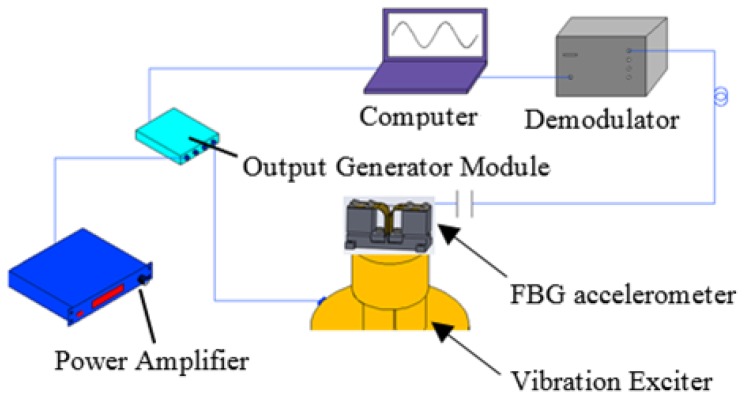
Experimental setup of the acceleration sensing system.

Based on the above-mentioned optimized structural parameters, we fabricated an accelerometer sensor prototype. As shown in Figure 4, instruments including the output generator module (Type 3160-A-042, Brüel & Kjær Sound & Vibration Measurement, Nærum, Denmark), the power amplifier (Type 2719, B&K, Nærum, Denmark) and the vibration exciter (Type 4808, B&K, Nærum, Denmark) were used to test the accelerometer sensor. The output generator module was used to adjust the parameters of the vibration signal. The frequency range of the vibration exciter is 5 Hz~10 kHz. In addition, we developed the demodulator and its software. The fiber grating was fabricated by UV (Ultra Violence) laser irradiation, and its initial center wavelength is 1532.51 nm. The FBG accelerometer is fixed on a shake table, and the signals of the FBG reflective wavelength are sent to an optical sensing interrogator. In our experiments, room temperature was 25 °C. The central wavelength shift of the FBG depended on the excitation acceleration.

**Figure 5:**
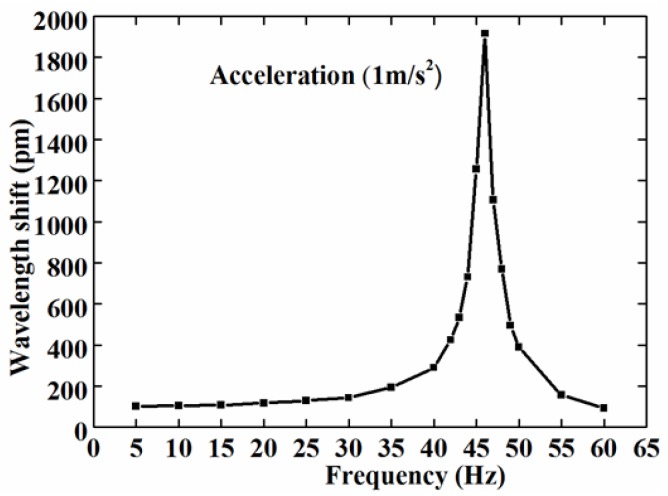
The frequency response of the accelerometer under acceleration of 1 m/s^2^.

We firstly observed the response of the accelerometer in the frequency domain. Giving the shake table a constant sinusoidal acceleration with an amplitude of 1 m/s^2^, and an excitation frequency from 5 Hz to 60 Hz, we were able to acquire the data of the FBG wavelength shifts from the interrogator. Figure 5 shows the frequency response curve of the FBG accelerometer.

From Figure 5, it can be seen that the variation of the wavelength shifts was constant within range of 5~20 Hz. However, when the excitation frequency was within the 20~46 Hz range, the wavelength shift steeply increased and reached the peak at 46 Hz. Between 46–60 Hz, the central wavelength shift was sharply decreased. As expected, the measured eigenfrequency was close to the theoretical calculation value of 51 Hz. It can be determined that the eigenfrequency of the sensor is 46 Hz, and the working frequency band is 5–20 Hz. Generally, the maximum operating frequency is approximately one-third to one-half of the eigenfrequency. So we can infer that the experimental results matched the expectations.

**Figure 6:**
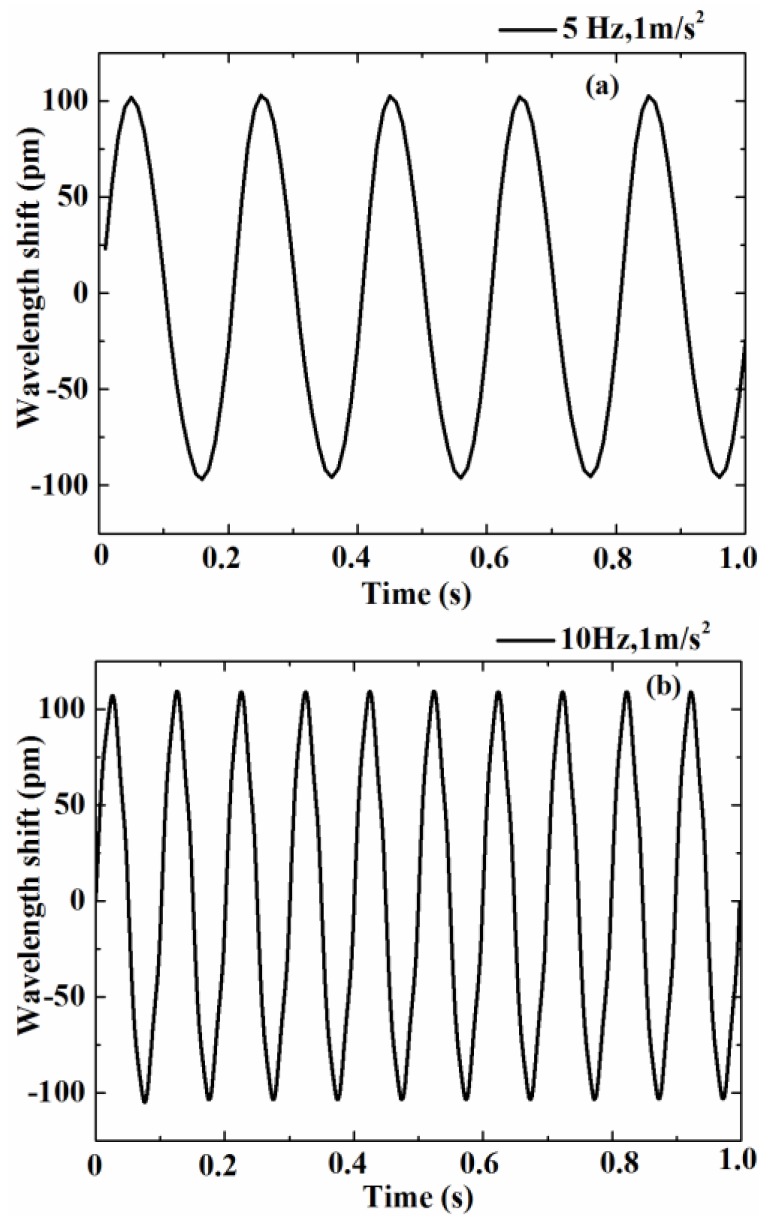
Time domain waveform of the sensor at 5 Hz (**a**) and 10 Hz (**b**).

After determining the working frequency, the setting acceleration and the excitation frequency of the exciter, the vibration waveforms in the time domain graph were obtained when the acceleration was 1 m/s^2^ and excitation frequency was 5 Hz or 10 Hz, as shown in Figure 6. It can be seen from the figure that the time domain waveforms had a good response to the external excitation in the work band. In the practical applications, the frequency spectrum can be obtained by Fourier transform from the time domain waveforms. Then the frequency information of the measured vibration body can be analyzed.

**Figure 7:**
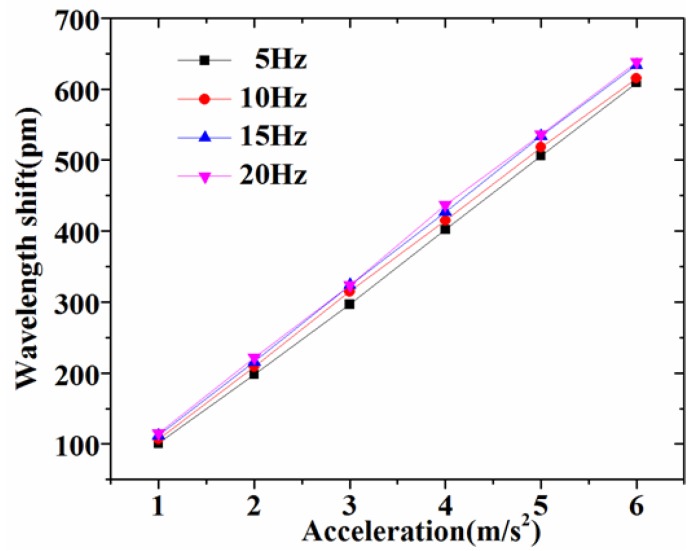
Responses of the sensor under different excitation accelerations.

**Figure 8:**
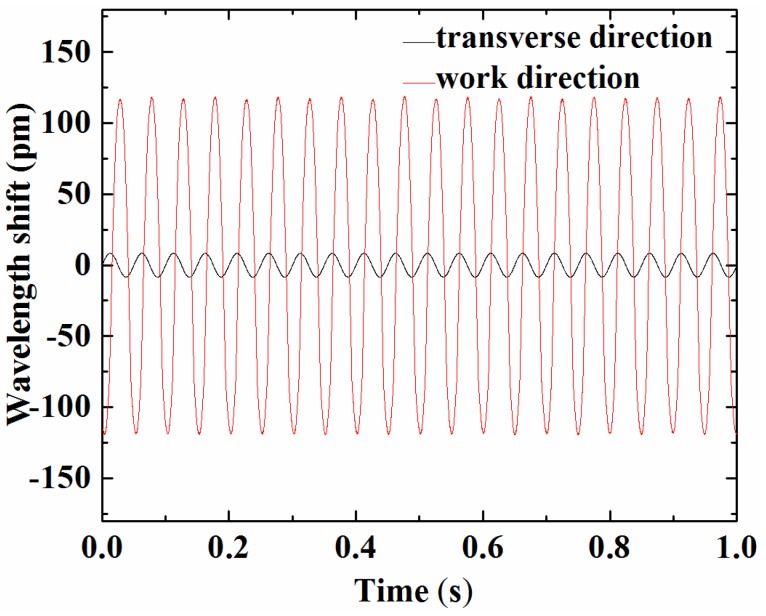
Cross-sensitivity of the sensor: response under excitation acceleration of 1 m/s^2^ and frequency at 20 Hz.

Secondly, we experimentally evaluated the response sensitivity of the accelerometer at different excitation frequencies (5 Hz, 10 Hz, 15 Hz, 20 Hz) and excitation accelerations varying from 1–6 m/s^2^, as shown in Figure 7. It can be seen that the FBG accelerometer demonstrated fairly good linearity related to the applied acceleration. Through the slope of the straight line, we estimated the sensitivity as 1067 pm/g at 5 Hz, 1084 pm/g at 10 Hz, 1126 pm/g at 15 Hz and 1166 pm/g at 20 Hz, respectively. From Figure 6 and Figure 8, it can be seen that the response sensitivity of the accelerometer had little correlation with the excitation frequency. For practical use, we must implement the calibration of the sensor based on the shake table test results.

Thirdly, we also observed the cross-sensitivity of the sensor. The exciter was rotated 90° to make its direction of vibration perpendicular to the sensitive direction of the sensor. As a result, sensor was applied with horizontal vibration. At the same time, we demodulated the shift of the central wavelength of the sensor with the optical fiber grating dynamic wavelength demodulator. With the amplitude of the excitation signal held at 1 m/s^2^ and the excitation frequency set at 20 Hz, the wavelength response in the work and transverse directions was demonstrated as shown in Figure 8. As a result, we realized that the transverse response amplitude is about 6.9% of the work direction amplitude. The prototype FBG accelerometer has a preferable anti-interference capacity.

**Figure 9:**
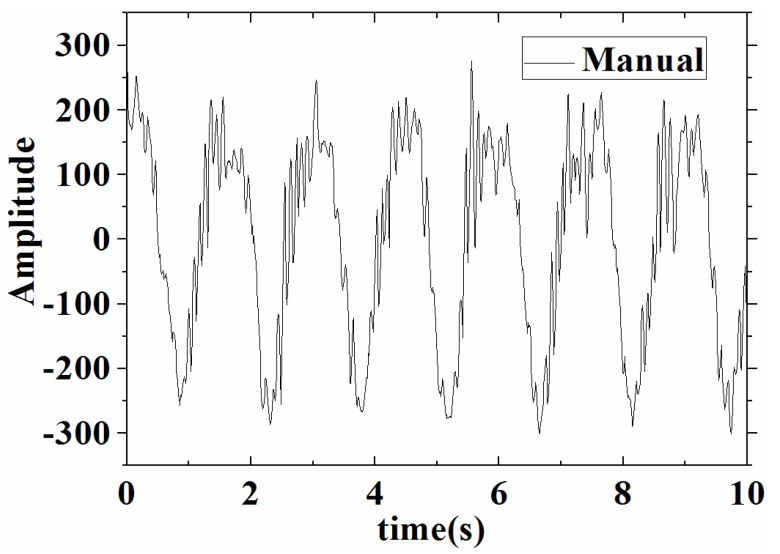
Vibration time domain waveform with manual simulation.

On the other hand, in our experiment, the vibration test system recorded a minimum frequency of 5 Hz. Due to the frequency limit of the vibration test system, a simple manually simulated vibration experiment was employed below 5 Hz. In the manual mode, we made the base of the sensor move up and down at a lower frequency, while the frequency and amplitude of the motion remained fixed. Ultimately, we realized the vibration time domain waveform, as shown in Figure 9.

It can be noted from Figure 9 that the time domain waveform of the acceleration sensor was basically intact. It showed that the low-frequency acceleration sensor responded to a low-frequency manual simulation vibration signal. Meanwhile, some clutter and jitter signals appeared in the waveform due to the shaking of the hand and the incoherence of the movement. Again, in Figure 9, the frequency spectrum graph was obtained by Fourier transform and generated a vibration frequency of about 0.7 Hz.

## 6. Conclusions

This paper proposes a novel architecture for a low-frequency FBG accelerometer sensor, whose sensitivity is highly improved and the eigenfrequency reduced. Geometrically, the sensor consists of a fiber grating, two inertia masses, and two symmetrical spring plates which act as elastic elements.

By numerical simulation, the influence of the structural parameters on the sensitivity and the eigenfrequency were revealed. Based on the simulation results, we discussed how to choose the structural parameters in order to realize balance between the sensitivity and the eigenfrequency.

Finally, a prototype accelerometer with an eigenfrequency near 46 Hz was designed and fabricated. The shake table test results showed that the sensor can operate in a low-frequency range from 0.7 Hz to 20 Hz with a response sensitivity of about 1067 pm/g. This accelerometer sensor is endowed with a higher sensitivity and can be used in some low-frequency applications, such as vibration monitoring of bridges.
